# The yellow hairy tongue

**DOI:** 10.11604/pamj.2018.30.298.16328

**Published:** 2018-08-30

**Authors:** Aryé Weinberg, Andreas Eberhard Albers

**Affiliations:** 1 Prosper-Hospital, Department of Otorhinolaryngology, Head and Neck Surgery, Recklinghausen, Germany; 2 Department of Otorhinolaryngology, Head and Neck Surgery, Berlin Institute of Health, Charité-Universitätsmedizin Berlin, Freie Universität Berlin, Humboldt-Universität zu Berlin, Campus Benjamin Franklin, Berlin 12200, Germany

**Keywords:** Hairy tongue, oral hygiene, smoking, alcohol, coffee

## Image in medicine

A 36-year-old woman presented to our out-patient clinic because she noticed that her tongue turned partially hairy, however she suffered from no other condition. She has been smoking 25 cigarettes a day for the last 17 years. Clinical exam showed a soft yellowish tongue with a hairy center. The rest of the clinical exam was normal. Otherwise the aero-digestive tract examination revealed no other pathology. A hairy tongue is a common benign clinical condition caused by proliferation of the papillae facilitating the collection of bacteria and cellular debris that finally lead to a yellow discoloration that may vary from black, brown to yellow. Contributing factors are smoking, excessive consumption of coffee, alcohol, poor oral hygiene, hyposalivation, eating a soft diet and the use of certain antibiotics such as tetracyclines. The treatment consists of the correction of the contributing factors. In our case the cessation of smoking and the improvement of oral hygiene was recommended resulted in full recovery.

**Figure 1:**
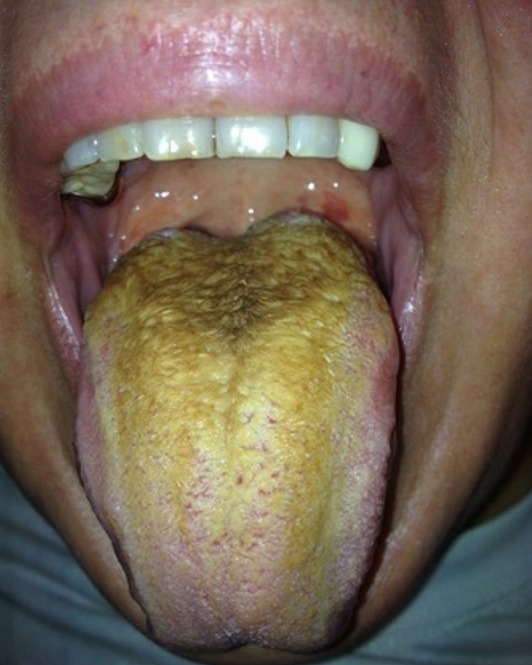
Hairy yellow tongue

